# Research progress on related mechanisms of uric acid activating NLRP3 inflammasome in chronic kidney disease

**DOI:** 10.1080/0886022X.2022.2036620

**Published:** 2022-04-05

**Authors:** Miao Wang, Xin Lin, Xiaoming Yang, Yanlang Yang

**Affiliations:** a Department of Nephrology, Yijishan Hospital of Wannan Medical College, Wuhu, China

**Keywords:** Uric acid, NLRP3 inflammasomes, organelle, oxidative stress

## Abstract

Hyperuricemia is an independent risk factor for the progression of chronic kidney disease. High levels of uric acid can lead to a series of pathological conditions, such as gout, urinary stones, inflammation, and uric acid nephropathy. There is a close relationship between uric acid and the NLRP3 inflammasome. NLRP3 inflammasome activation can cause cell damage and even death through endoplasmic reticulum stress, lysosome destruction, mitochondrial dysfunction, and the interaction between the Golgi apparatus and extracellular vesicles. In addition, the NLRP3 inflammasome acts as a molecular platform, triggering the activation of caspase-1 and the lysis of IL-1β, IL-18 and Gasdermin D (GSDMD) through different molecular mechanisms. Cleaved NT-GSDMD forms pores in the cell membrane and triggers pyrophosphorylation, thereby inducing cell death and releasing many intracellular proinflammatory molecules. In recent years, studies have found that hyperuricemia or uric acid crystals can activate NLRP3 inflammasomes, and the activation of NLRP3 inflammasomes plays an important role in kidney disease. This article reviews the possible pathophysiological mechanisms by which uric acid activates inflammasomes and induces kidney damage at the cellular and molecular levels.

## 1. Introduction

Uric acid (UA) is the final product of purine nucleotide metabolism. Under physiological conditions, it has a redox effect to maintain homeostasis in the body. Hyperuricemia is closely related to metabolic syndrome, diabetes, hypertension, and kidney and cardiovascular diseases [1]. The kidneys play an important role in regulating serum uric acid levels. Abnormal uric acid levels may affect kidney function. In chronic kidney disease (CKD), the serum uric acid concentration increases to pathological levels, which may lead to renal tubular damage, endothelial dysfunction, oxidative stress and intrarenal inflammation [1,2]. A number of recent studies have shown that uric acid can affect the morphology and function of renal parenchymal cells by activating the NLRP3 inflammasome and secreting related inflammatory factors. After the cells are stressed, various organelles in the cell become dysfunctional and participate in the NLRP3 inflammasome activation process, thereby affecting the occurrence and development of CKD in hyperuricemia [3,4]. The intracellular environment of a cell is essential for maintaining cell function and many cellular processes. Organelle damage caused by endogenous factors or invading pathogens has been shown to activate NLRP3 inflammasomes, leading to proinflammatory cytokine release and cell death induced by cell pyrophosphorylation. The role of pathological concentrations of uric acid or uric acid crystals in this organelle damage is still unclear, and the exact mechanism by which the NLRP3 inflammasome is activated by organelle damage remains to be elucidated. Here, we summarize the cellular and molecular mechanisms by which uric acid may be involved in the activation of NLRP3 inflammasomes and provide new ideas for the pathogenesis of hyperuricemia in nephropathy.

## 2. The physiological properties of uric acid

Most uric acid is excreted in urine through the kidneys. If the blood uric acid level is too high, urate crystals can easily form, which can lead to gout and urinary stones [5]. Under physiological conditions, the production and excretion of uric acid in the body maintain a dynamic balance. When certain pathogenic factors affect the body's homeostasis (For example, excessive intake of purine-containing foods, primary or secondary kidney disease lead to decreased glomerular filtration capacity), it will cause excessive production or insufficient excretion of uric acid, resulting in hyperuricemia, and triggering a series of metabolic disorders [6]. Uric acid is an essential antioxidant in the body, and it is involved in the body's antioxidant system to resist various pathogenic factors [7]. However, uric acid exposure can increase oxidative stress in endothelial cells, proximal tubule epithelial cells, mesangial cells, adipocytes and other cells [8]. Oxidative stress is closely related to the activation of the NLRP3 inflammasome, which provides a new pathogenesis theory for the occurrence and development of hyperuricemia in chronic kidney disease.

## 3. Understanding the NLRP3 inflammasome and its involvement in cell pathogenesis

The NLRP3 inflammasome is an important defensive component of the body's innate immune and stress system, and its role in kidney disease has also attracted much attention in recent years.

### 3.1. Structure and function of the NLRP3 inflammasome

The NLRP3 protein molecule is composed of three domains: PYD (pyridine domain), NACHT (central nucleotide-binding oligomerization domain), and LRR (leucine-rich repeat domain). NLRP3 is recruited through PYD-PYD interactions. The adaptor protein ASC (apoptosis-related dot-like protein) promotes the recruitment of the effector protein pro-caspase-1 (the precursor of cysteine protease 1) to form an inflammatory complex [9]. In summary, the NLRP3 inflammasome is a multicomponent assembly of adaptor and effector proteins that are highly expressed in myeloid cells, including NLRP3, adaptor protein apoptosis-related dot-like protein (ASC) and caspase-1 [9,10]. The inflammasome can recruit and activate the proinflammatory protease Caspase-1 by recognizing pathogen-related molecular patterns (PAMPs) or host-derived danger signal molecules (DAMPs). Activated Caspase-1 cleaves the precursors of IL-1 and IL-18, produces corresponding mature cytokines, and triggers a series of subsequent inflammatory reactions [10].

### 3.2. Activation of the NLRP3 inflammasome

The NLRP3 inflammasome is activated in various autoinflammatory and autoimmune diseases (such as gout, rheumatoid arthritis and systemic lupus erythematosus) [11]. In macrophages, the activation of NLRP3 can be divided into two signals: the initiation signal and the activation signal. The first step, usually Toll-like receptors (TLR) activation, triggers the NF-kappaB (NF-kB) pathway to regulate the gene transcription of pro-IL-1β and pro-IL-18 [12]. These inflammatory cytokines are released into the cytoplasm in the form of IL-1β and IL-18 precursors, which have no inflammatory biological activity. The activation signal in the second step leads to the assembly of the NLRP3-ASC inflammasome complex, the oligomerization of the NLRP3 molecule through its NACHT domain, the recruitment of ASC to form inflammasomes, the activation of caspase 1, and finally the cleavage and secretion of proinflammatory cytokines to form mature inflammatory factors with inflammatory effects [13]. However, in other types of cells (such as endothelial cells and podocytes), pro-IL-1 and other related molecules, such as ROS (reactive oxygen species), may directly activate NLRP3 inflammasomes [14]. The second step of NLRP3 inflammasome activation can be achieved by excessive stimulation by different toxins, from particulate matter to bacterial toxins.

### 3.3. Activator of NLRP3 inflammasome

Previous studies have shown that there are many activators of the NLRP3 inflammasome, including ATP, K + efflux, lysosomal function, endoplasmic reticulum (ER) stress, intracellular calcium overload, ubiquitination, microRNA, cathepsin B, etc. [13]. The most critical factor is the production of reactive oxygen species (ROS). In addition to mtROS, mitochondrial DNA (mtDNA) has also been reported to directly cause NLRP3 inflammasome activation. In addition, nonmicrobial particulate matter, such as alum, asbestos, silica, uric acid, etc., can also stimulate the activation of NLRP3 inflammasomes [10]. Uric acid crystals (MSU) or soluble uric acid can cause the activation of NLRP3 inflammasomes [15]. However, research on the specific mechanism of hyperuricemia and NLRP3 inflammasome activation is not clear. This article will summarize the relevant mechanisms at the cellular and molecular levels.

## 4. The relevant mechanism of the activation of the NLRP3 inflammasome by uric acid at the cellular level

### 4.1. Mitophagy

Mitochondria are dynamic organelles that continuously fuze and divide. Mitochondria clear damaged mitochondria through mitochondrial division and autophagy to maintain their own function and integrity. Autophagy helps to suppress inflammasomes and excessive inflammation [16]. The activation of autophagy can lead to the degradation of pre-IL-1β, thereby preventing the secretion of mature IL-1β [17]. Autophagy and inflammasome activation are two basic cellular responses to various stresses. The former leads to an effective lysosomal degradation pathway to eliminate large protein aggregates and damaged organelles, while the latter allows the molecular platform to activate caspase-1 and secrete IL-1β when exposed to dangerous stimuli [18]. Under normal physiological conditions, autophagy and inflammasomes form a dynamic balance to maintain homeostasis. Uric acid is a strong pro-oxidant in cells [19]. The cells in patients with hyperuricemia are very likely to have disrupted redox homeostasis and autophagy and inflammasome dynamics, causing further irreversible damage to the cells. However, there are still many questions about the molecular mechanism by which autophagy controls the activation of inflammasomes through multiple agonists in different situations. Mitochondrial quality control is a mechanism by which autophagy regulates the activation of inflammasomes. The key measure by which autophagy maintains cell homeostasis is to recognize and remove abnormal mitochondria through mitochondrial autophagy [20]. Under cytopathological conditions, autophagy can cause mitochondrial dysfunction, significantly increased mitochondrial reactive oxygen species (mtROS) production, and mitochondrial DNA translocation into the cytoplasm. These pathological processes may lead to the excessive activation of the NLRP3 inflammasome, causing subsequent cell damage [21]. Experiments have confirmed that in endothelial cells, uric acid can increase cellular oxidative stress by activating NADPH oxidase in the cytoplasm, and after UA treatment, the enzyme activity in isolated mitochondria is significantly reduced. Acutely high concentrations of UA can cause mitochondrial calcium overload in endothelial cells, reduce mitochondrial DNA copy number, significantly increase mitochondrial membrane potential, and cause excessive ROS production. Mitochondrial uncoupling occurs at the same time, reducing ATP synthesis [22]. In addition, cardiolipin, a phospholipid located in the inner mitochondrial membrane (IMM), is also involved in the activation of the NLRP3 inflammasome. NLRP3 is recruited to mitochondria during the initiation phase and binds to cardiolipin on the outer mitochondrial membrane (OMM). Studies have found that the movement of cardiolipin to the OMM depends on the ROS generated during the NLRP3 initiation step. Surprisingly, caspase-1 also binds to cardiolipin on the mitochondria, and ASC is only recruited after NLRP3 is activated [23]. There are abundant uric acid transporters and mitochondria in human renal tubular epithelial cells. Whether it can cause mitochondrial autophagy and further activate the NLRP3 inflammasome under the condition of hyperuricemia remains to be further elucidated.

### 4.2. Endoplasmic reticulum stress

The endoplasmic reticulum (ER) is a large intimal compartment that is highly sensitive to disturbances and is the center of many organelle network functions, which suggests that the ER may act as a relay station between the stressor and mitochondria, linking stress and inflammatory signals. NLRP3 has been shown to exist in the ER before its activation, and ER stress activates the NLRP3 inflammasome through caspase-1 [24]. The ER is a membrane-bound organelle. In addition to being a site for lipid synthesis and Ca2+ homeostasis, the ER is also essential for protein folding, assembly and modification. Under endoplasmic reticulum stress conditions, NLRP3 may promote the release of mitochondrial contents through the cysteine protease caspase-2. This leads to the release of mtDNA and cytochrome c into the cytoplasm [24]. The ER is also the location where Ca2+ homeostasis occurs, and Ca2+ mobilization is related to the activation of the NLRP3 inflammasome [25]. The ER is not only involved in lipid synthesis but is also involved in the perception and regulation of intracellular cholesterol levels. It has recently been demonstrated that blocking cholesterol efflux from lysosomes can inhibit the activation of NLRP3 inflammasomes in mouse macrophages, which is due to a decrease in ER cholesterol [26]. Similarly, the biosynthesis of cholesterol in the ER is inhibited by exposing cells grown in lipoprotein-deficient media to statins, thereby inhibiting the activation of the NLRP3 inflammasome [26]. Therefore, cholesterol may directly affect the formation of inflammasome complexes, or ER cholesterol content can support NLRP3 to achieve the necessary conformation. In any case, these studies emphasize the key role of ER in the activation of inflammasomes. Hyperuricemia model rats can cause cardiomyocyte apoptosis, interstitial fibrosis, diastolic dysfunction and increased endoplasmic reticulum (ER) stress, and allopurinol treatment can alleviate these changes [27]. In endothelial cells, uric acid can induce the production of reactive oxygen species, enhance PKC-dependent nitric oxide synthase (eNOS) phosphorylation and mediate endoplasmic reticulum stress [28]. MSU crystals can induce human fibroblast-like synovial cells to release ROS and reactive nitrogen species (RNS) and then oxidize proteins and change the cell oxidation state of the endoplasmic reticulum, leading to apoptosis [28]. Recently, important findings about the activation mechanism of the NLRP3 inflammasome induced by MSU and crystalline silica in human macrophages have been reported. Under resting conditions, ASC is located in the mitochondria, cytoplasm and nucleus, while NLRP3 is located in the ER. NLRP3 inflammasome activators (such as MSU) induce mitochondrial damage and then accumulate acetylated α-tubulin. Then, using microtubules, acetylated α-tubulin mediates the transfer of ASC-containing mitochondria to the ER-bearing region in a dynein-dependent manner, resulting in an increase in the colocalization of ASC and NLRP3, thereby activating NLRP3-mediated inflammasomes [29]. Interestingly, the use of colchicine inhibits the transport of ASC to NLRP3 in the production of the ER and IL-1β. These findings show that microtubules mediate the assembly and activation of NLRP3 inflammasomes by MSU and silica [29].

### 4.3. Golgi apparatus

In recent years, the role of the Golgi apparatus in the activation of NLRP3 inflammasomes has gradually been recognized. The Golgi body contains many different enzymes, including glycosyltransferase, oxidoreductase, phosphatase, and protein kinase, which provide conditions for the activation and assembly of the NLRP3 inflammasome. In a recent study, brefeldin A disrupted Golgi integrity and reduced caspase-1 activation, IL-1β secretion and ASC spot formation after NLRP3 inflammasome activation [30], suggesting that Golgi activation and NLRP3 activation are closely related. NLRP3 is recruited to the endoplasmic reticulum membrane associated with mitochondria (MAM) and is activated by MAM-derived effectors. MAM is located near the Golgi membrane. After stress, the diacylglycerol (DAG) level in the Golgi apparatus increases rapidly, recruiting the key effector protein of DAG, protein kinase D (PKD). The phosphorylation of NLRP3 by PKD on the Golgi apparatus is sufficient to release NLRP3 from MAM, leading to the assembly of active inflammasomes [31].High concentrations of uric acid or uric acid crystals activate NLRP3 inflammasomes and can cause intracellular oxidative stress and endoplasmic reticulum dysfunction. However, few studies have investigated whether uric acid can change the recruitment, assembly and displacement of NLRP3 inflammasomes by affecting changes in the endoplasmic reticulum-Golgi cell inner membrane system.

### 4.4. Extracellular vesicles

In prokaryotes and eukaryotes, extracellular vesicles (EVs) are recognized as effective carriers of communication between cells. This is due to their ability to transfer proteins, lipids and nucleic acids, thereby affecting various physiological and pathological functions of recipient cells and parent cells [32]. During the immune response, EVs selectively load immunomodulatory proteins, such as cytokines, which makes them important messengers for transmitting targeted biological signals. Vesicle signaling has now become an important part of innate immunity, which coordinates the role of multiple immune system cells in infectious and inflammatory diseases. EV secretion has been associated with inflammation for a long time. Various experiments have proven that classic NLRP3 activators (calcium oxalate and sodium urate (MSU) crystals, ATP, β-glucan, and viral RNA) and nonclassical caspase-4 activation can induce EV and EV-related proteins [33,34]. These signals, processes and mechanisms link the activation of NLRP3 inflammasomes with EV-mediated secretion of proinflammatory cytokines and other immunomodulatory proteins. The NLRP3 inflammasome is activated in the cytoplasm of cells, and its products (such as IL-1β) are exported through the nonclassical ER-Golgi pathway, mainly through secretory lysosomes and microvesicle (MV) and extracellular vesicle (EV) exocytosis. Studies have found that increased D-ribose in the kidney may act on podocytes, not only inducing the activation of NLRP3 inflammasomes but also increasing the level of ceramide in lysosomes, thereby promoting the secretion of EVs and transporting NLRP3 inflammatory products from podocytes to play a role in triggering glomerular inflammation [35]. Regarding the process of uric acid-mediated NLRP3 inflammasome activation, the role of EVs requires additional experimental evidence to prove.

### 4.5. Pyrolysis

Pyrolysis, also known as cell inflammatory necrosis, is a kind of programmed cell death mediated by Gasdermin protein and is an important natural immune response of the body. When the cell is stimulated by the outside, the cytoplasmic inflammasome is usually specifically detected by both homocaspase activation and the interaction between the recruitment domain (CARD) and the apoptosis-related dot-like protein (ASC) of the adaptor protein and responds to host and pathogen-derived dangerous molecules to recruit and activate caspase-1. Caspase-1 is then cleaved to activate the cytokines IL-1β and IL-18 to drive the inflammatory response, and the activated GSDMD N-domain is released by cleaving the pyrophosphate cell death effector Gasdermin D (GSDMD) to form pores in the cell membrane, causing cell lysis and death. There is evidence that sodium urate crystals can mediate the activation of GSDMD by activating the NLRP3 inflammasome, causing pyrolysis [36].

## 5. The activation pathway of NLRP3 inflammasome in uric acid nephropathy and its potential pathogenic mechanism

**Figure 1.**
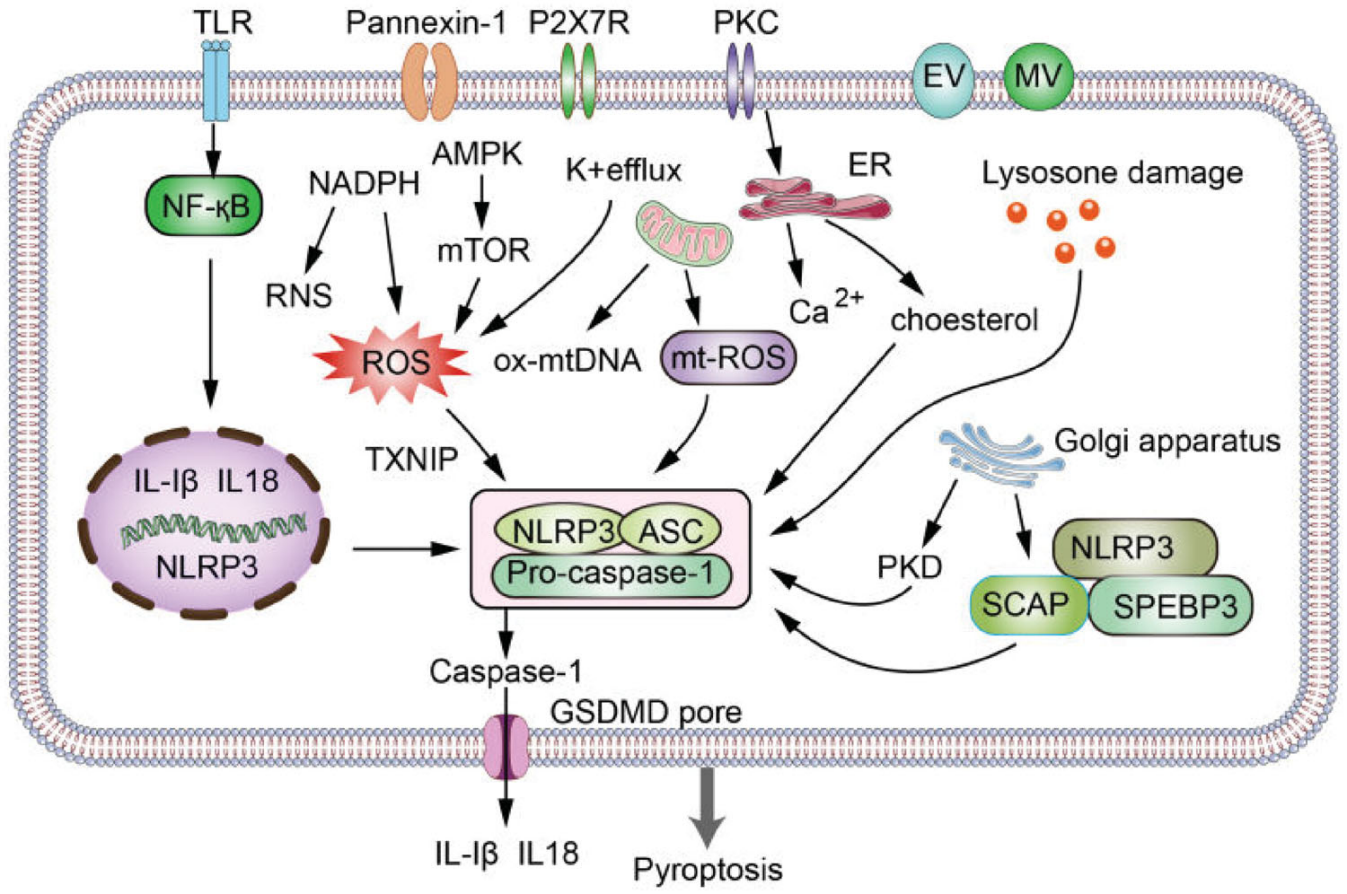
Uric acid activates the NLRP3 inflammasome through oxidative stress, potassium efflux, mitochondrial changes, lysosome destruction, endoplasmic reticulum stress, Golgi transport and other pathways.

**Figure 2.**
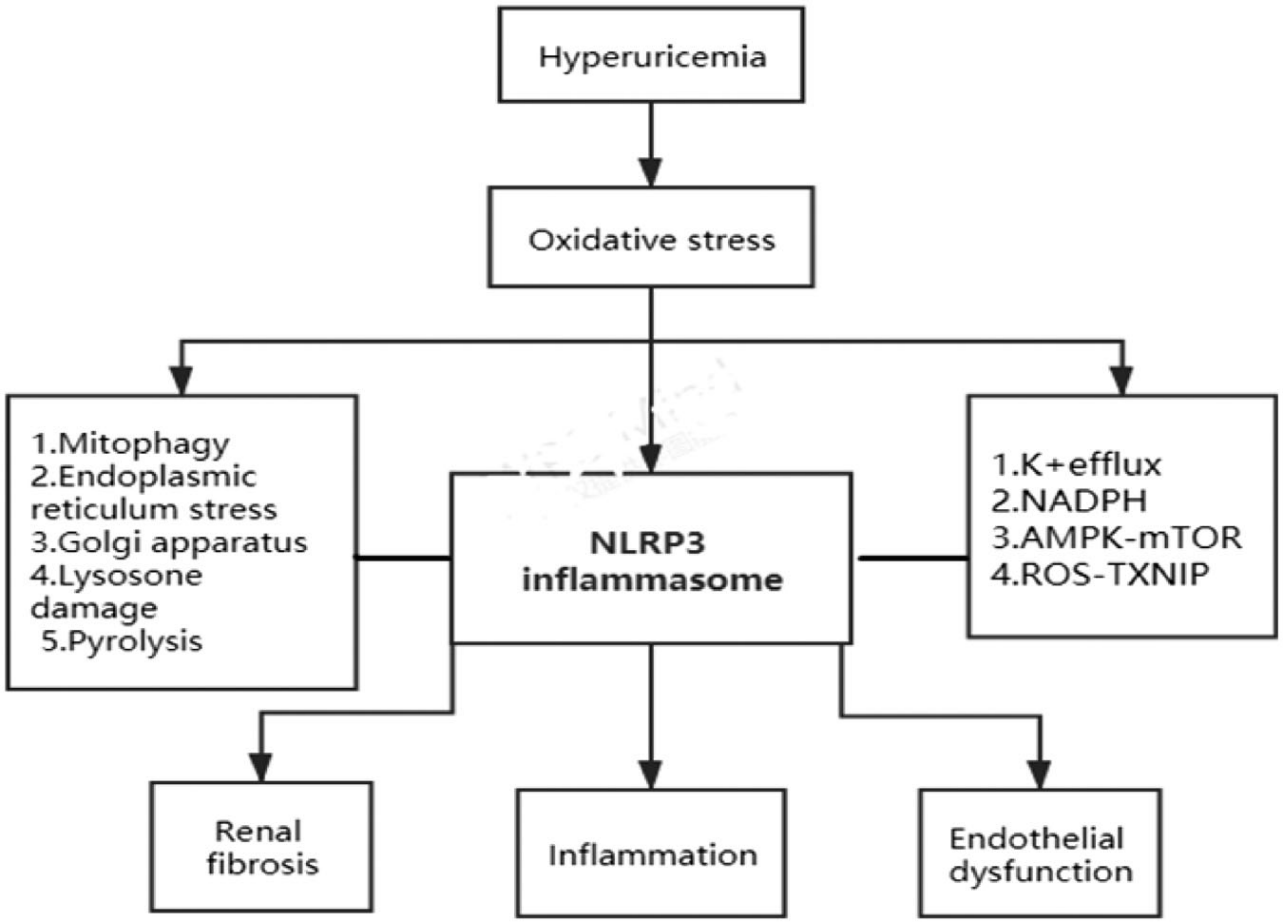
The mechanisms by which hyperuricemia mediates NLRP3 inflammasome-induced kidney damage mainly include oxidative stress, endothelial dysfunction, inflammation, and renal fibrosis.

Hyperuricemia is closely associated with chronic kidney disease. Uric acid can affect the morphology and dysfunction of renal parenchymal cells by activating the NLRP3 inflammasome and secreting related inflammatory factors [37]. After the cells are stressed, various organelles in the cell become dysfunctional and participate in the NLRP3 inflammasome activation process, thereby affecting chronic the occurrence and development of kidney disease (Figure 1). Activation of NLRP3 in kidney disease exacerbates the inflammatory response and subsequent fibrosis [38]. Studies have shown that uric acid can cause the proliferation of vascular endothelial cells through the activation of NLRP3 inflammasome, and induce the accumulation of α-smooth muscle actin (α-SMA) [15]. The activated NLRP3 inflammasome stimulates the secretion of IL-1β and IL-18 and causes severe kidney damage. IL-1β and IL-18 are major inflammatory cytokines. They can induce the formation of foam in renal vascular endothelial cells, the accumulation of inflammatory cells on the vessel wall, the proliferation and vasodilation of smooth muscle cells, and IL-1β recruits neutrophils to release other pro-inflammatory cytokines [39]. In addition, the NLRP3 inflammasome activated by uric acid is associated with epithelial-mesenchymal transition (EMT), vascular endothelial cell damage and tubulointerstitial fibrosis [40]. Recently, mROS was confirmed related to the CKD, and silencing the NLRP3 gene could alleviate the associated mitochondrial dysfunction and renal fibrosis [41]. Aldosterone can directly damage the renal tubule, which is a key point in CKD pathogenesis. Kadoya *et al.* showed that high level of aldosterone could activate the inflammasomes *via* mROS [42]. Up-regulation of NLRP3 mRNA in kidney biopsy of patients with various kidney diseases (including IgA nephropathy, acute tubular necrosis, focal segmental glomerulosclerosis, lupus nephritis, minimal change disease and hypertensive nephropathy) has been confirmed [43]. With the increasing incidence of hyperuricemia, hyperuricemia not only increases the risk of disease, but also affects the prognosis of kidney disease. The mechanisms by which hyperuricemia mediates NLRP3 inflammasome-induced kidney damage mainly include oxidative stress, endothelial dysfunction, inflammation, and renal fibrosis (Figure 2). However, the entire mechanism of kidney damage caused by hyperuricemia is complex and not yet fully understood, so further research is needed. Based on previous studies, we can conclude that uric acid induces the activation of NLRP3 inflammasomes and causes kidney damage mainly through the following pathways:

### 5.1. Uric acid releases ROS through mitochondrial changes to activate NLRP3

Under disease conditions, changes in mitochondrial morphology and function are closely related to NLRP3 activation. Mitochondria are an important source of ATP required for cell function, but once the mitochondria are damaged, they produce too many stress signals, leading to cell dysfunction and ultimately to programmed cell death. The generation of ROS is obviously the most important trigger for NLRP3 activation [44]. ROS can be used as a redox signaling molecule to activate or regulate the activation of the NLRP3 inflammasome, but the specific mechanism is still unclear [45]. The main source of ROS is the oxidative phosphorylation that occurs in the mitochondrial respiratory electron transport chain. Mitochondrial damage caused by various external stimuli may release excessive ROS, thereby affecting the activation of NLRP3 inflammasomes [46]. In addition to mtROS, mitochondrial DNA, especially oxidized mitochondrial DNA, causes mitochondrial dysfunction, resulting in the release and activate NLRP3 inflammasomes [22]. Uric acid can stimulate NADPH oxidase 4 (NOX4), release free radicals, and induce mitochondria to produce excessive ROS in a series of oxidative stress responses in the hydrophobic environment of cells, triggering the activation of NLRP3 inflammasomes [47–49]. Mitochondrial damage can cause NLRP3 oligomerization or induce α-tubulin acetylation by providing reactive oxygen species (ROS), relocating mitochondria to the vicinity of NLRP3 and acting upstream of NLRP3 activation. In addition, mitochondria themselves also contribute to the activation of inflammasomes, and mitochondria can be used as a signal platform for the assembly of inflammasome proteins [50]. Under stimulation by an NLRP3 activator, mitochondrial antiviral signal protein (MAVS) or mitochondrial fusion protein (mitofusin 2, Mfn2) may be involved in the recruitment and assembly of NLRP3 inflammasomes. However, the specific mechanism by which hyperuricemia causes mitochondrial damage in renal tubular epithelial cells is still unclear, and a large number of experiments and clinical studies are needed to prove this hypothesis.

### 5.2. Uric acid activates NLRP3 through the ROS-TXNIP pathway of cellular oxidative stress

In the process of chronic kidney disease, the inflammation process and immune cell stimulation lead to an increase in free radicals and reactive oxygen species and a decrease in antioxidant capacity, which leads to oxidative stress. Uric acid can regulate the NLRP3/IL-1β signaling pathway through ROS activation and further induce the damage of vascular endothelial cells in the early stage of CKD [4]. In addition to the mitochondria mentioned above, ROS can also be produced by inducing the activation of NADPH oxidase in the cytoplasm [51]. In addition, uric acid stimulates the RAS system to produce additional ROS in cells, which can cause cellular oxidative stress [52]. Oxidative stress caused by increased uric acid levels is an independent risk factor for kidney damage. Mitochondrial damage caused by oxidative stress has become a hot research topic in recent years. Uric acid interferes with rat renal tubular epithelial cells, affects the structure and function of mitochondria, and triggers the apoptosis of renal tubular epithelial cells by stimulating oxidative stress [53]. Mitochondria are the main organelles damaged in epithelial cells through oxidative stress [54]. The increase in ROS concentration after cell stress leads to the separation of thioredoxin-interacting protein (TXNIP) from oxidized thioredoxin 1 (Trx-1), and then TXNIP binds to NLRP3 to activate the NLRP3 inflammasome [44, 55]. TXNIP is an important substance that associates oxidative stress with NLRP3 inflammasomes [56]. MSU can induce the activation of NLRP3 inflammasomes through the TXNIP-mediated NF-κB signaling pathway and intracellular TXNIP translocation [55]. In addition, the crystal structure of NLRP3 contains a highly conserved disulfide bond that connects the PYD and the nucleotide-binding site domain, which is highly sensitive to oxidative stress [55].

### 5.3. Uric acid activates the NLRP3 inflammasome by increasing potassium outflow

After uric acid stimulates renal tubular epithelial cells, excessive ROS production will increase cell K + efflux and further activate NLRP3 inflammasomes [4,57]. According to previous studies, the activation of ligand-gated ion channels in the purinergic receptor family, such as K+/H + carriers and ATP-mediated purinergic receptor 7 (P2X7), promotes the maturation of IL-1β by increasing K + excretion [58]. In addition, ATP is a strong activator of the NLRP3 inflammasome, which can reduce the cytoplasmic K + concentration by 50% [4].

### 5.4. Uric acid activates the NLRP3 inflammasome through the AMPK-mTOR-ROS pathway

AMPK (AMP-activated protein kinase) is a key regulatory pathway of cell energy metabolism. Serum uric acid can regulate AMPK-mTOR (mammalian target of rapamycin)-mitochondrial reactive oxygen species, and the HIF-1α (hypoxia inducible factor-1α) pathway mediates the enhancement of the inflammatory process [59]. A decrease in uric acid levels will lead to the activation of AMPK to reduce inflammation [60]. Hyperuricemia will further cause inflammation, autophagy, and mitochondrial dysfunction through damage to sodium-potassium pump signal transduction and eventually lead to cell damage. The AMPK-mTOR pathway is abundant in renal tubular epithelial cells [52]. Hyperuricemia can stimulate the activation of AMPK in proximal tubular epithelial cells, in this study, they found that UA stimulates AMPK activity as a protective mechanism; however, soon AMPK activity decreases, leading to the impairment of Na+-K+-ATPase signaling, which further triggers inflammation autophagy, and mitochondrial dysfunction and leads to cell injury. Sustained treatment with an AMPK activator significantly alleviated UA-induced alterations [61].

### 5.5. Uric acid activates the NLRP3 inflammasome through the TLR/NF-KB pathway

When stimulated by uric acid and uric acid crystals, monocytes and macrophages activate cell membrane Toll-like receptors (TLRs) and Nod-like receptors (NLRs). These receptors can initiate major innate immune pathways, such as nuclear factor ĸB (NF-ĸB) and NLRP3 inflammasomes, which cause metabolic and phenotypic changes in immune cells and parenchymal cells and trigger the secretion of various inflammatory mediators, which may cause irreversible tissue damage and loss of function [62]. Recent studies on the TLR family have found that TLR2 and TLR4 are closely related to the activation of inflammasomes by uric acid. Uric acid can stimulate TLR4 to increase NFKB transcription and further activate inflammasomes, release interleukin-1, and increase inflammation [12]. Soluble uric acid enhances NALP3 expression in proximal renal tubular epithelial cells in a TLR4-dependent manner and increases caspase-1 activation and IL-1β and ICAM-1 (cell adhesion factor) production, suggesting the activation of innate immunity in human primary renal proximal tubular epithelium cells [40].

## 6. Conclusion

The NLRP3 inflammasome is activated by hyperuricemia and responds to DAMPs (including ROS, ATP, extracellular matrix components and crystalline substances) released from damaged kidney tissue. From the cellular level to the molecular level, the related mechanisms involved are numerous and complex. Although the activation mechanism and the involvement of internal kidney cells and immune cells have yet to be determined, in many kidney diseases, the inflammasome has already damaged the kidneys. New evidence in the study of the NLRP3 inflammasome may contribute to the development of renal autoimmunity in kidney disease.
